# Never‐breastfed children face a higher risk of suboptimal cognition at 2 years of corrected age: A multinational cohort of very preterm children

**DOI:** 10.1111/mcn.13347

**Published:** 2022-03-16

**Authors:** Carina Rodrigues, Jennifer Zeitlin, Michael Zemlin, Emilija Wilson, Pernille Pedersen, Henrique Barros, Effective Perinatal Intensive Care in Europe (EPICE) Research Group

**Affiliations:** ^1^ EPIUnit, Instituto de Saúde Pública Universidade do Porto Porto Portugal; ^2^ CRESS, Obstetrical, Perinatal and Pediatric Epidemiology Research Team (EPOPé) INSERM, INRA, Université de Paris Paris France; ^3^ Department of Neonatology and Pediatrics University Children's Hospital of Saarland Homburg Germany; ^4^ Department of Women's and Children's Health Karolinska Institutet Stockholm Sweden; ^5^ Department of Neonatology Hvidovre Hospital Hvidovre Denmark

**Keywords:** breastfeeding, breast milk, cognitive development, neurodevelopment, very preterm children

## Abstract

In a cohort of children born very preterm (VPT), we investigated the association between breast milk feeding (BMF) initiation and its duration on cognitive development at 2 years of corrected age. Data were obtained from the Effective Perinatal Intensive Care in Europe population‐based prospective cohort of children born <32 weeks of gestation, in 11 European countries, in 2011–2012. The study sample included 4323 children. Nonverbal cognitive ability was measured applying the Parental Report of Children's Abilities, except for France where the problem‐solving domain of the Ages & Stages Questionnaire was used. Verbal cognition was based on the number of words the child could say. To determine the association between BMF (mother's own milk) and nonverbal and verbal cognition (outcome categorized as optimal and suboptimal), adjusted risk ratios (aRRs) were estimated fitting Poisson regression models, with inverse probability weights to account for nonresponse bias. Overall, 16% and 11% of the children presented suboptimal nonverbal and verbal cognition, respectively. Never BMF was associated with a significantly increased risk for suboptimal nonverbal (aRR = 1.29, 95% confidence interval [CI] = 1.09–1.53) and verbal (aRR = 1.45, 95% CI = 1.09–1.92) cognitive development compared with those ever breastfed, after adjustment for perinatal and sociodemographic characteristics. Compared with children breastfed 6 months or more, children with shorter BMF duration exhibited a statistically nonsignificant elevated aRR. VPT children fed with breast milk had both improved nonverbal and verbal cognitive development at 2 years in comparison with never breastfed, independently of perinatal and sociodemographic characteristics. This study encourages targeted interventions to promote BMF among these vulnerable children.

## Key messages

- In a European cohort of children born very preterm, 16% and 11% presented suboptimal nonverbal and verbal cognitive development, respectively.
- Never‐breastfed children faced a higher risk of having suboptimal nonverbal and verbal cognition at 2 years of corrected age when compared with those who were breastfed, independently of perinatal and sociodemographic characteristics.
- Breastfeeding support is a modifiable factor regardless of perinatal and sociodemographic characteristics, which reinforces the importance of specifically targeted interventions to protect, promote and support breast milk feeding in neonatal intensive care units and after discharge.

## Graphical abstract

In a European cohort of children born very preterm, we investigated the association between breast milk initiation and its duration on cognitive development at 2 years of corrected age. We found that never‐breastfed children faced a higher risk of having suboptimal nonverbal and verbal cognition at 2 years of corrected age when compared with those who were breastfed, independently of perinatal and sociodemographic characteristics.

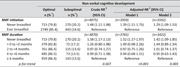

## 1 INTRODUCTION

There is a solid evidence base showing that breast milk feeding (BMF) in healthy, full‐term infants improves cognitive abilities in childhood, adolescence, and adulthood (Horta et al., 2015; Victora et al., 2015). A meta‐analysis of 17 observational studies reported that BMF was associated with higher scores in intelligence tests (+3.4 intelligence quotient [IQ] points; Horta et al., 2015). This positive effect was also observed in a large cluster‐randomized trial designed to evaluate a BMF promotion intervention, suggesting that the association is causal: the experimental group had a 5.9 higher cluster‐adjusted mean difference in the full‐scale IQ (Kramer et al., 2008).

The presence of biological components in breast milk, such as long‐chain polyunsaturated fatty acids (especially docosahexaenoic acid and arachidonic acid) and human milk oligosaccharides, essential for the infant's brain development, might explain the association, as these components play an important role in neurogenesis and modulate infant gut microbiota composition, respectively (Agostoni, 2008; Belfort, 2018; Innis, 2004; Mayer et al., 2014). Growing evidence supports the existence of a microbiota–gut–brain axis, in which the microbiome is thought to interact with the brain through immunological, endocrine, and neural pathways, although the exact mechanisms are not yet clear (Keunen et al., 2015). Another potential mechanism is related to the children's emotional environment. Mothers who breastfeed may provide stronger attachment security and be more sensitive to the needs of the infant (Peñacoba & Catala, 2019; Tharner et al., 2012), which have been associated with brain development and better neurocognitive outcomes (Treyvaud et al., 2016; Wang et al., 2019). Furthermore, BMF can also be an indirect indicator of other parenting behaviours, which are independently associated with cognition (Horta et al., 2015), as higher maternal education and socioeconomic status are positively associated with the prevalence and duration of BMF in high‐income countries (Victora et al., 2016). Thus, the link between BMF and cognitive development may therefore be partly confounded by family and parenting characteristics (Horta et al., 2015).

Very preterm (VPT < 32 weeks of gestation) children experience a higher risk of short‐term and long‐term adverse health and developmental outcomes, including cerebral palsy, motor and cognitive delay (Ancel et al., 2015; Pierrat et al., 2017), and require more healthcare services (Seppänen et al., 2019). The importance of BMF on their neurodevelopment might be even more significant considering that VPT children are particularly vulnerable to nutrient deficits and growth faltering, presenting very specific nutritional needs compared with full‐term infants (Belfort, 2018; El Rafei et al., 2020; Koletzko et al., 2018). At the same time, preterm neonatal morbidities which affect cognition, such as brain lesions, may operate through pathways that may not be affected by the nutritional or immunological benefits of BMF. Because of these specificities, studies based on full‐term children cannot be generalized to this population (Twilhaar et al., 2018).

The evidence about the relationship between BMF and neurodevelopment in VPT infants is inconclusive, being an area of ongoing research (Koo et al., 2014; Lechner & Vohr, 2017; Miller et al., 2018). Some observational studies found an independent positive association between BMF and neurodevelopment (Beaino et al., 2011; Belfort et al., 2016; Gibertoni et al., 2020; Johnson et al., 2011; Rozé et al., 2012), whereas others found none (Furman et al., 2004; Jacobi‐Polishook et al., 2016; Pinelli et al., 2003). One randomized clinical trial of 363 very low birthweight (VLBW) infants found that the use of supplemental donor milk compared with preterm formula, as a supplement to mother's milk during hospitalization, did not improve neurodevelopment at 18 months' corrected age (CA; O'Connor et al., 2016), raising further questions about the differences between donor human milk and mother's own milk (Hård et al., 2019). Moreover, it remains unclear if prolonged BMF for more than 6 months has an influence on the neurodevelopmental outcome in VPT.

Recent systematic reviews and meta‐analysis based on children born VPT and/or VLBW revealed that different methodological approaches such as population inclusion criteria (according to gestational age and/or birthweight), age at assessment, BMF indicators/definitions (e.g., exclusive vs. any BMF; BMF considered during hospital stay, at or after hospital discharge; and different cutoffs for the duration), methods and instruments for assessing and classifying neurodevelopment, the cognitive abilities considered as outcome, as well as design limitations (e.g., sample size) make a direct comparison between studies difficult (Koo et al., 2014; Lechner & Vohr, 2017; Miller et al., 2018; Sentenac, Boutron, et al., 2020).

Taking into account the inconsistent findings and also the lack of data on the total duration of BMF in this vulnerable population, we aimed to investigate the association of BMF (mother's own milk) initiation and its duration with neurodevelopment, specifically on nonverbal and verbal cognition at 2 years of CA in a large prospective cohort of VPT children from 11 European countries.

## 2 METHODS

### 2.1 Data source

This study used data from the Effective Perinatal Intensive Care in Europe (EPICE) project, which recruited a geographically defined population‐based prospective cohort of VPT infants (22–31 completed weeks of gestational age), born in 19 regions from 11 European countries (Zeitlin et al., 2020). As previously described, data were collected during 12 consecutive months in 2011–2012, starting between March and July 2011 (Zeitlin et al., 2020), except for the three French regions where inclusions lasted 6 months as part of the study Etude Epidémiologique sur les Petits Ages Gestationnels (EPIPAGE)‐2 (Ancel et al., 2015).

Data on maternal, obstetric and infant characteristics, and on the neonatal course were retrieved from obstetrical and neonatal patient records using a pretested standardized questionnaire with common definitions filled in by medical staff until discharge from the hospital and later verified by the research team. At 2 years of CA, data on child's health and neurodevelopment outcomes, growth, health service utilization, BMF and sociodemographic characteristics were collected using a structured questionnaire completed by parents. This questionnaire was translated into the different national languages, backtranslated in English and pretested in all the regions (Zeitlin et al., 2020).

### 2.2 Study population

Of the 7900 VPT live births identified, 6792 infants were discharged alive (86.0%). Thirty‐one infants died before 2 years CA, leaving 6761 eligible infants for follow‐up at 2 years CA, and parents provided information for 4426 children (65.5%). As the response rate in the Northern Region in the United Kingdom was very low (27.2%, 103/379), this region was excluded from this analysis because of concern about a potentially biased sample, in accordance with other studies at 2 years CA in this cohort (Bonnet et al., 2019; Seppänen et al., 2019). After excluding the UK Northern region, follow‐up rates differed across countries, ranging from 47.2% in Belgium to 99.3% in Estonia. The final study sample included 4323 children (67.7% of those eligible; Figure S1).

### 2.3 Data on BMF (exposure)

Data on BMF was collected through the parental questionnaire at 2 years CA, considering only mother's own milk, independently of route of administration (nasogastric tube, bottle and/or directly at the breast). Infants were considered to have ever been breastfed if the answer was yes to the question ‘Was your child breast fed?’. BMF duration was defined as the age of the child, reported in months of chronological age, at which BMF was stopped completely, regardless of if it was exclusive or partial. We categorized the BMF duration into five groups: (1) never breastfed; (2) between >0 and <2 months; (3) between 2 and <4 months; (4) between 4 and <6 months; and (5) 6 months or more.

### 2.4 Data on nonverbal and verbal cognitive development (outcome)

Regarding our outcome of interest, nonverbal and verbal cognitive abilities were measured at 2 years CA through a validated developmental assessment tool filled in by parents, the Parent Report of Children's Abilities‐Revised (PARCA‐R), which includes the short version of the MacArthur Communicative Development Inventory for language abilities (Johnson et al., 2004, 2008). This tool has shown excellent test–retest reliability and correlation with the Mental Development Index of the Bayley Scales of Infant and Toddler Development‐II (Cuttini et al., 2012; Johnson et al., 2008). The full PARCA‐R, which includes a nonverbal and verbal score, could not be completed in countries without a translation of the MacArthur short form available (Belgium, Denmark, the Netherlands, Poland, Portugal and Sweden; Zeitlin et al., 2020). Therefore, we additionally included questions on language acquisition, which were asked in all regions (Does your child say as many as 10 words?; Does your child say as many as 50 words?; Has your child started to combine words into short sentences yet? (e.g., Mummy up, Daddy gone, I do it). The Ages & Stages Questionnaire (ASQ) was used in French regions as this instrument was validated in France, whereas the PARCA‐R was not (Flamant et al., 2011; Zeitlin et al., 2020). The problem‐solving component of the ASQ score was used as its content is closest to the PARCA‐R, as done in previous studies in the EPICE cohort (El Rafei et al., 2021; Wolf et al., 2021).

For this study, we categorized both non‐verbal and verbal cognition as a binary outcome, optimal and suboptimal. For nonverbal cognitive development, we established a cutoff of <22 for the nonverbal cognition score of the PARCA‐R and <29.78 for the ASQ problem‐solving domain, which corresponds to <2 SDs below the mean of the standardization sample to define suboptimal nonverbal cognition (Johnson et al., 2008). A negative response to the following question ‘Does your child say as many as 10 words?’ was considered as suboptimal verbal cognition, as this is an indicator of very low expressive vocabulary (<5th percentile) (Fenson et al., 2000).

### 2.5 Data on perinatal and sociodemographic characteristics (covariates)

Perinatal and sociodemographic characteristics were used to describe the sample and as covariates in the analyses. We included child sex, gestational age (in completed weeks), small for gestational age (SGA) defined as birthweight <10th percentile for gestational age (severe SGA as <3rd percentile and moderate SGA as 3rd to <10th percentile) using intrauterine references developed for the cohort (Zeitlin et al., 2017), any congenital anomaly (none and nonsevere vs. severe), bronchopulmonary dysplasia (BPD) defined as oxygen dependency or respiratory support at 36 weeks postmenstrual age, Intraventricular Haemorrhage Papille's grades III or IV (IVH III–IV), cystic periventricular leukomalacia (cPVL), Retinopathy of Prematurity grades III‐V (ROP III–V), and necrotizing enterocolitis (NEC) with surgery or peritoneal drainage. As defined previously for this cohort, we used a perinatal risk composite, considering three risk groups based on perinatal characteristics, which represents the overall risk of health and developmental problems at discharge from the neonatal unit (Seppänen et al., 2019) as follows: (1) Lower risk—birth at 30–31 weeks of gestation, not SGA, no congenital anomaly and without severe neonatal morbidities (BPD, IVH III–IV, cPVL, ROP III–V, NEC with surgery or peritoneal drainage); (2) Moderate risk—birth at 28–29 weeks' gestation or SGA, or any nonsevere congenital anomaly, but no severe neonatal morbidities; and (3) Higher risk—birth before 28 weeks of gestation or at least one severe neonatal morbidity (BPD, IVH III–IV, cPVL, ROP III–V, NEC with surgery or peritoneal drainage) or severe congenital anomaly.

We adjusted for CA at assessment (in months), categorized in three categories (<22 months CA, 22–26 months CA, and >26 months CA). The CA corresponds to the chronological age (when the questionnaire was filled by parents) minus the number of weeks that infant was born early. For example, a child with 24 months of chronological age who was born 3 months (12 weeks) early would have a CA of 21 months. Using infant's CA is particularly important for the development outcomes in children born VPT to give an accurate assessment of the developmental abilities and is recommended in the assessment of VPT development at 2 years of age (Gould et al., 2021).

We assessed maternal and sociodemographic characteristics, including mother's age at delivery (≤24, 25–34, or ≥35 years), mother's country of birth (native‐born, born in Europe and born outside Europe as in the UK data were available only on ethnicity), family situation at 2 years CA (mother living with partner vs. single caregiver or other family situation), parity at delivery and type of pregnancy (singleton vs. multiple). We also included the mother's highest achieved educational level collected at the 2 years CA based on the International Standard Classification of Education (ISCED) 2011 definition and categorized in three groups, considering the cross‐national differences in educational systems: (1) lower level (ISCED Level 0–2: lower secondary); (2) intermediate level (ISCED Level 3–5: upper or postsecondary, nontertiary or short cycle tertiary); and (3) higher level (ISCED Level 6–8: bachelor degree or higher; UNESCO Institute for Statistics, 2012).

### 2.6 Statistical analysis

Responders and nonresponders' characteristics were compared considering all eligible infants for follow‐up at 2 years CA, excluding infants from UK Northern region (*n* = 6382). We calculated the Wald's test from the logistic regression adjusted for country because of differences in follow‐up rates. The effects of potential bias due to attrition were accounted for using the inverse probability weighting method in all analyses to give higher weight to children with characteristics of non‐responders to obtain a sample that reflects the full set of observations (Bonnet et al., 2019; Seaman & White, 2013; Seaman et al., 2012). The probability of follow‐up at 2 years CA (yes or no) was estimated with a multivariate logistic regression model including all variables available at baseline associated with nonresponse, as detailed elsewhere (Bonnet et al., 2019; Seppänen et al., 2020). The weight was computed using the total sample of eligible infants for follow‐up at 2 years CA (*n* = 6382). Before predicting the inverse probability weights, missing values from variables included in the regression model were imputed with multiple chained equations (20 complete data sets generated) to have a more stable weight (Seaman & White, 2013; Seaman et al., 2012). Data were assumed to be missing at random. All results presented in this study are based on weighted sample.

BMF initiation, as well as nonverbal and verbal cognitive development were described by the study sample characteristics and proportions compared performing Wald's test from the logistic regression adjusted for country, entered as a categorical variable. To estimate the association between BMF and nonverbal and verbal cognition (optimal vs. suboptimal), crude risk ratios (RR) and adjusted RRs (aRRs) with 95% confidence intervals (95% CIs) were estimated by fitting multilevel mixed‐effects generalized linear regression models with a log link, Poisson distribution and robust SEs. This model directly estimates the RRs and provides covariate aRRs and associated SEs for binary outcomes (Zou & Donner, 2013). RRs were preferred to odds ratios as suboptimal cognition is not a rare event in VPT infants (incidence of 10% or more) and our study used a prospective cohort design. We used multilevel models with random intercepts at the country and mother levels to consider the correlation of infants within countries and between siblings, respectively. To determine whether there is a dose–response relationship by BMF duration, we performed a test for trend.

Variables selected for the adjusted models were based on the scientific literature and previous findings from the EPICE cohort (Bonnet et al., 2019; Draper et al., 2020; Sentenac, Johnson, et al., 2020; Wilson et al., 2018), including characteristics that were related or plausibility related with both BMF and cognitive development (potential confounders). In addition, stratified analyses were performed to assess the association of BMF with nonverbal and verbal cognition by perinatal risk group (as an indicator of the overall risk of health and developmental problems) and maternal educational level (as an indicator of socioeconomic disadvantage), comparing the total crude RRs to the stratum‐specific RRs.

Statistical analyses were performed using STATA version 15.1 software (Stata Corporation).

## 3 RESULTS

Characteristics associated with loss to follow‐up at 2 years CA are provided in detail in Table S1. Children born from singleton pregnancies and to younger, multiparous and migrant women were less likely to be followed up. Children who received BMF at discharge were more likely to participate at 2 years CA.

Table 1 shows the infant and maternal characteristics of ever breastfed children (weighted distributions). We observed that proportions of never‐breastfed children were higher for younger, multipara, single and lower educational level mothers.

**Table 1:** Any BMF, according to the infant, neonatal morbidity, maternal and socio‐demographic characteristics

	Any breast milk feeding (*n* = 4305)		
	Never (*n* = 952; 23.1%)	Ever (*n* = 3353; 76.9%)	
Characteristics	*n* (%)^a^	*n* (%)^a^	*p* b
Infant and neonatal morbidity			
Child sex			
Male	504 (23.0)	1775 (77.0)	0.872
Female	448 (23.1)	1578 (76.9)	
Gestational age at birth			
22–25 Weeks	84 (26.1)	239 (73.9)	0.007
26–27 Weeks	192 (27.2)	563 (72.8)	
28–29 weeks	238 (20.7)	906 (79.3)	
30–31 Weeks	438 (22.5)	1645 (77.5)	
SGA			
<3rd Percentile	213 (23.9)	683 (76.1)	0.704
3rd to <10th Percentile	115 (24.4)	396 (75.6)	
≥10th Percentile	624 (22.6)	2274 (77.4)	
BPD			
No	781 (22.2)	2897 (77.8)	<0.001
Yes	142 (27.1)	388 (72.9)	
ROP Stages III–V			
No	883 (22.5)	3213 (77.5)	<0.001
Yes	60 (39.0)	99 (61.0)	
IVH III–IV/cPVL			
No	871 (22.9)	3133 (77.1)	0.234
Yes	70 (25.6)	187 (74.4)	
NEC needing surgery or peritoneal drainage			
No	931 (23.0)	3308 (77.0)	0.037
Yes	21 (30.6)	45 (69.4)	
Any congenital anomaly			
None	850 (22.5)	3093 (77.5)	0.023
Nonsevere	81 (27.1)	231 (72.9)	
Severe	21 (46.6)	28 (53.4)	
Perinatal risk			
Lower	222 (21.5)	907 (78.5)	<0.001
Moderate	326 (20.3)	1301 (79.7)	
Higher	379 (27.4)	1062 (72.6)	
Mother and sociodemographic characteristics			
Mother's age at delivery			
≤24 Years	159 (29.3)	373 (70.7)	<0.001
25–34 Years	508 (21.2)	1998 (78.8)	
≥35 Years	282 (23.6)	975 (76.4)	
Parity at delivery			
0 Previous births	508 (20.0)	2078 (80.0)	<0.001
1 Previous birth	248 (24.8)	792 (75.2)	
2 Or more previous births	181 (29.5)	454 (70.5)	
Type of pregnancy			
Singleton	680 (24.8)	2201 (75.2)	<0.001
Multiple	272 (19.3)	1152 (80.7)	
Mother's country of birth			
Native‐born	714 (22.9)	2563 (77.1)	0.134
Born elsewhere in Europe	51 (31.0)	147 (69.0)	
Born outside Europe	131 (22.0)	449 (78.0)	
Mother's educational level			
Low level (ISCED 0–2)	278 (34.7)	558 (65.3)	<0.001
Intermediate level (ISCED 3–5)	391 (22.6)	1366 (77.4)	
High level (ISCED 6–8)	214 (14.3)	1347 (85.7)	
Family situation			
Mother living with partner	812 (22.0)	3026 (78.0)	<0.001
Single mother or other family situation	102 (28.8)	280 (71.2)	
Country (region[s])			
Belgium (Flanders)	59 (21.7)	246 (78.3)	<0.001
Denmark (Eastern)	10 (6.2)	170 (93.8)	
Estonia (whole country)	19 (13.8)	119 (86.2)	
France (Burgundy, Northern, Ile‐de‐France)	357 (37.2)	629 (62.8)	
Germany (Hesse, Saarland)	111 (30.7)	319 (69.3)	
Italy (Emilia, Lazio, Marche)	183 (28.1)	549 (71.9)	
The Netherlands (East‐Central)	29 (14.1)	199 (85.9)	
Poland (Wielkopolska)	69 (36.8)	130 (63.2)	
Portugal (Lisbon, Northern)	52 (15.1)	350 (84.9)	
Sweden (Stockholm)	7 (5.0)	156 (95.0)	
UK (East Midlands, Yorkshire and Humber)^c^	56 (14.3)	486 (85.7)	

Abbreviations: BMF, breast milk feeding; BPD, bronchopulmonary dysplasia; CA, corrected age; cPVL, cystic periventricular leukomalacia; ISCED, International Standard Classification of Education 2011; IVH, intraventricular haemorrhage; NEC, necrotizing enterocolitis; ROP, retinopathy of prematurity; SGA, small for gestational age.

^a^ Proportions were calculated using inverse probability weights to account for nonresponse bias.

^b^ Wald's test from logistic regression adjusted for country.

^c^ UK Northern region was excluded from this analysis.

Of the 4092 children with available information, 16.0% presented suboptimal nonverbal cognitive development (weighted sample). This proportion varied among countries, with the lowest rate found in the Netherlands (10.6%) and the highest in Poland (24.8%; Table 2). For verbal cognition, 11.1% (*n* = 4134) of the children presented suboptimal ability (weighted sample) and this proportion ranged from 7.7% in Sweden to 19.1% in Poland (Table 2).

**Table 2:** Nonverbal and verbal cognitive development, according to infant, neonatal morbidity, maternal and socio‐demographic characteristics

	Nonverbal cognition (*n* = 4092)			Verbal cognition (*n* = 4134)		
	Optimal (*n* = 3474; 84.0%)	Suboptimal (*n* = 618; 16.0%)		Optimal (*n* = 3691; 89.9%)	Suboptimal (*n* = 443; 11.1%)	
Characteristics	*n* (%)^a^	*n* (%)^a^	*p* b	*n* (%)^a^	*n* (%)^a^	*p* b
Infant and neonatal morbidity						
Child sex						
Male	1775 (81.1)	388 (18.9)	<0.001	1868 (85.9)	300 (14.1)	<0.001
Female	1699 (87.4)	230 (12.6)		1823 (92.3)	143 (7.7)	
Gestational age at birth						
22–25 Weeks	229 (72.3)	83 (27.7)	<0.001	244 (76.8)	68 (23.2)	<0.001
26–27 Weeks	576 (80.0)	128 (20.0)		599 (84.2)	108 (15.8)	
28–29 Weeks	930 (83.8)	165 (16.2)		1016 (91.0)	93 (9.0)	
30–31 Weeks	1739 (87.3)	242 (12.7)		1832 (91.3)	174 (8.7)	
SGA						
<3rd Percentile	697 (80.9)	155 (19.1)	0.013	727 (84.5)	126 (15.5)	<0.001
3rd to <10th Percentile	425 (86.1)	66 (13.9)		442 (89.9)	47 (10.1)	
≥10th Percentile	2352 (84.6)	397 (15.4)		2522 (90.1)	270 (9.9)	
BPD						
No	3025 (85.5)	473 (14.5)	<0.001	3207 (90.4)	328 (9.6)	<0.001
Yes	368 (73.0)	135 (27.0)		399 (78.2)	110 (21.8)	
ROP Stages III–V						
No	3334 (85.7)	556 (14.3)	<0.001	3529 (89.5)	402 (10.5)	<0.001
Yes	100 (64.5)	55 (35.5)		117 (73.9)	39 (26.1)	
IVH III–IV/cPVL						
No	3286 (85.8)	524 (14.2)	<0.001	3464 (89.9)	387 (10.1)	<0.001
Yes	153 (59.4)	87 (40.6)		188 (74.7)	51 (25.3)	
NEC needing surgery or peritoneal drainage						
No	3433 (84.3)	595 (15.7)	<0.001	3638 (89.0)	432 (11.0)	0.251
Yes	41 (66.3)	23 (33.7)		53 (83.8)	11 (16.2)	
Any congenital anomaly						
None	3207 (84.6)	544 (15.4)	0.025	3407 (89.5)	383 (10.5)	0.004
Nonsevere	231 (78.0)	63 (22.0)		248 (83.0)	49 (17.0)	
Severe	35 (77.4)	11 (22.6)		35 (78.3)	11 (21.7)	
Perinatal risk						
Lower	970 (89.9)	104 (10.1)	<0.001	1023 (93.4)	72 (6.6)	<0.001
Moderate	1348 (86.0)	208 (14.0)		1431 (91.2)	138 (8.8)	
Higher	1064 (76.7)	296 (23.3)		1138 (83.3)	228 (16.7)	
Corrected age at assessment						
≤21 Months	41 (83.9)	8 (16.1)	<0.001	44 (80.7)	11 (19.3)	0.052
22–26 Months	3053 (83.1)	579 (16.9)		3211 (88.8)	389 (11.2)	
≥27 Months	366 (92.8)	29 (7.2)		419 (90.6)	43 (9.4)	
Mother and socio‐demographic characteristics						
Mother's age at delivery						
≤24 Years	398 (82.4)	81 (17.6)	0.556	427 (86.6)	66 (13.4)	0.518
25–34 Years	2027 (83.9)	363 (16.1)		2167 (89.9)	244 (10.1)	
≥35 Years	1043 (85.3)	172 (14.7)		1092 (89.5)	128 (10.5)	
Parity at delivery						
0 Previous births	2131 (85.8)	340 (14.2)	<0.001	2252 (90.3)	241 (9.7)	0.010
1 Previous birth	830 (83.6)	158 (16.4)		880 (88.1)	119 (11.9)	
2 Or more previous births	476 (78.4)	117 (21.6)		522 (86.7)	80 (13.3)	
Type of pregnancy						
Singleton	2297 (83.2)	436 (16.8)	0.075	2461 (89.9)	296 (11.1)	0.923
Multiple	1177 (85.7)	182 (14.3)		1230 (89.0)	147 (11.0)	
Mother's country of birth						
Native‐born	2681 (84.6)	467 (15.4)	0.139	2888 (90.1)	308 (9.9)	0.003
Born elsewhere in Europe	162 (84.4)	28 (15.6)		156 (87.5)	26 (12.5)	
Born outside Europe	430 (81.1)	91 (18.9)		440 (84.4)	81 (15.6)	
Mother's educational level						
Low level (ISCED 0–2)	646 (82.4)	130 (17.6)	0.006	673 (85.1)	118 (14.9)	<0.001
Intermediate level (ISCED 3–5)	1420 (83.2)	268 (16.8)		1532 (89.9)	172 (10.1)	
High level (ISCED 6–8)	1316 (86.0)	200 (13.8)		1389 (91.3)	132 (8.7)	
Family situation						
Mother living with partner	3154 (84.5)	550 (15.5)	0.115	3345 (89.6)	384 (10.4)	0.024
Single mother or other family situation	297 (80.8)	59 (19.2)		319 (85.1)	51 (14.9)	
Country (region[s])						
Belgium (Flanders)	246 (80.9)	57 (19.1)	0.001	273 (90.1)	26 (9.9)	0.009
Denmark (Eastern)	152 (85.2)	27 (14.8)		162 (90.6)	17 (9.4)	
Estonia (whole country)	123 (89.1)	15 (10.9)		126 (91.3)	12 (8.7)	
France (Burgundy, Northern, Ile‐de‐France)	734 (86.2)	116 (13.8)		729 (86.7)	110 (13.3)	
Germany (Hesse, Saarland)	376 (86.6)	53 (13.4)		397 (90.5)	36 (9.5)	
Italy (Emilia, Lazio, Marche)	624 (84.9)	107 (15.1)		649 (89.1)	80 (10.9)	
The Netherlands (East‐Central)	206 (89.4)	21 (10.6)		205 (90.2)	20 (9.8)	
Poland (Wielkopolska)	149 (75.2)	48 (24.8)		162 (80.9)	37 (19.1)	
Portugal (Lisbon, Northern)	344 (85.0)	60 (15.0)		362 (91.6)	34 (8.4)	
Sweden (Stockholm)	130 (79.9)	33 (20.1)		151 (92.3)	13 (7.7)	
UK (East Midlands, Yorkshire and Humber)^c^	390 (81.1)	81 (18.9)		475 (87.7)	58 (12.3)	

Abbreviations: BPD, bronchopulmonary dysplasia; CA, corrected age; cPVL, cystic periventricular leukomalacia; ISCED, International Standard Classification of Education 2011; IVH, intraventricular haemorrhage; NEC, necrotising enterocolitis; ROP, retinopathy of prematurity; SGA, small for gestational age.

^a^ Proportions were calculated using inverse probability weights to account for nonresponse bias.

^b^ Wald's test from logistic regression adjusted for country.

^c^ UK Northern region was excluded from this analysis.

Suboptimal nonverbal and verbal ability at 2 years CA were more frequent in males, children born before 28 weeks of gestational age, and at higher perinatal risk (Table 2). When mothers were born outside Europe and with lower educational levels, the proportions of children with suboptimal non‐verbal and verbal cognition were higher.

As demonstrated in Table 3, never‐breastfed children faced a higher risk of suboptimal nonverbal cognition than those who had ever been breastfed (RR = 1.48, 95% CI: 1.11–1.98). Similarly, when stratified by perinatal risk group, never BMF was associated with an increased risk in the three groups, with a gradient from the lower to higher risk groups (Lower: RR = 1.02, 95% CI: 0.58–1.78; Moderate: RR = 1.21, 95% CI: 0.97–1.51; Higher: RR = 1.57, 95% CI: 1.12–2.18; Table S2). The stratified analysis by maternal educational level found that never BMF was associated with an increased risk of suboptimal non‐verbal cognition in all groups, with a significant association in the intermediate level group (RR = 1.55, 95% CI: 1.01–2.38; Table S3). Overall, after adjustment for sex, CA at assessment, and perinatal risk (Model 1), never BMF remained associated with an increased risk for suboptimal nonverbal cognition when compared with ever breastfed children (aRR = 1.39, 95% CI: 1.11–1.75; Table 3). Similarly, when adjusted only for sociodemographic characteristics (maternal age, parity, type of pregnancy, mother's country of birth, mother's educational level and family situation), without perinatal risk included in the model, children who were never breastfed presented 1.40 times more risk to have suboptimal non‐verbal cognition (95% CI: 1.13–1.72). This effect persisted after taking into consideration simultaneously perinatal, maternal and sociodemographic characteristics (aRR = 1.29, 95% CI: 1.09–1.53; Table 3, Model 2). The association between BMF duration and non‐verbal cognition was stronger among shorter duration categories when compared with infants breastfed until 6 months or more, with a dose–response relationship.

**Table 3:** Prevalence and RRs for nonverbal cognitive development, by BMF initiation and duration

	Nonverbal cognitive development				
	Optimal	Suboptimal	Crude RR^b^	Adjusted RR^b^ (95% CI)	
	*n* (%)^a^	*n* (%)^a^	(95% CI)	Model 1	Model 2
BMF initiation			(*n* = 4075)	(*n* = 3959)	(*n* = 3592)
Never breastfed	713 (79.8)	170 (20.2)	1.48 (1.11–1.98)	1.39 (1.11–1.75)	1.29 (1.09–1.53)
Ever breastfed	2749 (85.4)	443 (14.6)	Reference	Reference	Reference
BMF duration			(*n* = 3870)	(*n* = 3765)	(*n* = 3422)
Never breastfed	713 (79.8)	170 (20.2)	1.58 (1.17–2.13)	1.52 (1.17–1.97)	1.42 (1.09–1.85)
>0 to <2 Months	379 (82.8)	72 (17.2)	1.26 (0.80–1.98)	1.49 (0.98–2.26)	1.44 (0.89–2.34)
2 to <4 Months	761 (86.4)	115 (13.6)	0.97 (0.74–1.27)	0.97 (0.75–1.26)	1.01 (0.74–1.37)
4 to <6 Months	485 (86.5)	73 (13.5)	0.99 (0.71–1.38)	0.94 (0.68–1.31)	0.95 (0.63–1.43)
≥6 Months	959 (86.4)	143 (13.6)	Reference	Reference	Reference
*p*			0.007	<0.001	0.003

*Note*: Model 1: Adjusted for child sex, CA at assessment and perinatal risk. Model 2: Adjusted for child sex, CA at assessment, perinatal risk, maternal age, parity, type of pregnancy, mother's country of birth, mother's educational level and family situation.

Abbreviations: BMF, breast milk feeding (mother's own milk); CA, corrected age; CI, confidence interval; RR, risk ratio.

^a^ Proportions were calculated using inverse probability weights to account for nonresponse bias.

^b^ RRs were derived from the weighted sample (inverse probability of participating at the 2 years CA follow‐up), multilevel mixed‐effects generalized linear regression models with random intercepts at the country and mother level.

As shown in Table 4, we observed that children who were never breastfed presented 1.73 times more risk to have suboptimal verbal cognition (95% CI: 1.19–2.52). The stratified analysis by perinatal risk found that never BMF was associated with an increased risk of suboptimal verbal cognition in the three risk groups, with a significant association in the moderate risk group (RR = 2.17, 95% CI: 1.23–3.82; Table S2). In the same way, never BMF was also associated with an increased risk in all maternal educational level groups, with a significant association in both lower and intermediate groups (RR = 1.70, 95% CI: 1.02–2.82; RR = 1.76, 95% CI: 1.09–2.84, respectively; Table S3). Overall, this increased risk for never‐breastfed children remained significant after adjustment for sex, CA at assessment and perinatal risk (aRR = 1.59, 95% CI: 1.16–2.19; Table 4, Model 1). When adjusted only for sociodemographic characteristics (without perinatal risk included in the model), children who were never breastfed presented 1.59 times more risk to have suboptimal verbal cognition (95% CI: 1.13–2.24). After incorporating perinatal, maternal and sociodemographic characteristics (Model 2), significant differences persisted, even with slight changes in point estimates (aRR = 1.45, 95% CI: 1.09–1.92). We estimated crude RRs and aRRs considering the BMF duration and it was possible to verify a dose–response relationship (Table 4).

**Table 4:** Prevalence and RRs for verbal cognitive development, by BMF initiation and duration

	Verbal cognitive development				
	Optimal	Suboptimal	Crude RR^b^	Adjusted RR^b^ (95% CI)	
	*n* (%)^a^	*n* (%)^a^	(95% CI)	Model 1	Model 2
BMF initiation			(*n* = 4118)	(*n* = 3999)	(*n* = 3625)
Never breastfed	753 (84.3)	134 (15.7)	1.73 (1.19‐2.52)	1.59 (1.16‐2.19)	1.45 (1.09‐1.92)
Ever breastfed	2926 (90.4)	305 (9.6)	Reference	Reference	Reference
BMF duration			(*n* = 3894)	(*n* = 3787)	(*n* = 3440)
Never breastfed	753 (84.3)	134 (15.7)	1.78 (1.23–2.59)	1.65 (1.19–2.31)	1.50 (1.12–2.00)
>0 to <2 Months	416 (89.4)	47 (10.6)	1.18 (0.77–1.79)	1.42 (1.00–2.03)	1.21 (0.93–1.59)
2 to <4 Months	798 (91.3)	77 (8.7)	0.93 (0.78–1.10)	0.92 (0.76–1.13)	0.97 (0.74–1.26)
4 to <6 Months	516 (92.8)	43 (7.2)	0.80 (0.50–1.27)	0.72 (0.46–1.12)	0.80 (0.49–1.31)
≥6 Months	1010 (90.8)	100 (9.2)	Reference	Reference	Reference
*p*			0.017	0.005	0.011

*Note*: Model 1: Adjusted for child sex, CA at assessment and perinatal risk. Model 2: Adjusted for child sex, CA at assessment, perinatal risk, maternal age, parity, type of pregnancy, mother's country of birth, mother's educational level and family situation.

Abbreviations: BMF, Breast milk feeding (mother's own milk); CA, corrected age; CI, confidence interval; RR, Risk ratio.

^a^ Proportions were calculated using inverse probability weights to account for nonresponse bias.

^b^ RRs were derived from the weighted sample (inverse probability of participating at the 2 years CA follow‐up), multilevel mixed‐effects generalized linear regression models with random intercepts at the country and mother level.

A sensitivity analysis was carried out considering only children hospitalized in neonatal intensive care units (NICUs) without human bank milk/donor milk available. After adjustment for potential confounding factors, the risk was 1.70 (95% CI: 1.46–1.97) for nonverbal and 1.78 (95% CI: 1.49–2.14) for verbal suboptimal cognitive development, compared with those ever breastfed.

## 4 DISCUSSION

In a population‐based cohort of children born VPT in Europe, we found that 16% and 11% of the children presented suboptimal nonverbal and verbal cognitive development, respectively. Never‐breastfed children faced a higher risk to have suboptimal cognition than those who had ever been breastfed, independently of perinatal and sociodemographic characteristics. When considering BMF duration, we observed a dose–response relationship with higher risks among short durations compared with longer durations.

Our findings support previous research indicating that BMF has an independent association with neurodevelopment for VPT children (Beaino et al., 2011; Gibertoni et al., 2020; Johnson et al., 2011; Rozé et al., 2012). Two independent cohorts of VPT infants in France, Loire Infant Follow‐up Team and Etude Epidémiologique sur les Petits Ages Gestationnels (EPIPAGE), showed that any BMF at discharge was associated with a reduction in the odds of suboptimal neurodevelopment at 2 and at 5 years of age, respectively (Rozé et al., 2012). Also, in the EPIPAGE cohort, it was observed that any BMF at discharge was highly associated with lower odds of mild and severe cognitive deficiencies at 5 years (Beaino et al., 2011). Another study from the United Kingdom and Ireland, using data from the EPICure cohort, revealed that receiving any breast milk was a protective factor for reading scores at 11 years (Johnson et al., 2011). Recently, a study from Italy demonstrated that exclusively formula‐fed infants at discharge from the NICU had significantly lower neurodevelopment trajectories from 3 to 24 months of CA compared with those receiving exclusively human milk or mixed (Gibertoni et al., 2020). Data obtained from the United Kingdom Millennium cohort at 5 years showed a significant difference in mean score between children who were breastfed and who were never breastfed, after adjustment for potential confounders: in term children (≥37 weeks of gestation), a two‐point increase in score for picture similarities (breastfed ≥ 4 months) and naming vocabulary (breastfed ≥6 months); in preterm children (28–36 weeks of gestation), a four‐point increase for naming vocabulary (breastfed ≥4 months) and picture similarities (breastfed ≥2 months), and a six‐point increase for pattern construction (breastfed ≥2 months; Quigley et al., 2012).

The mechanisms linking BMF to neurodevelopment are still unclear, and even more so in VPT infants. Cognitive development is a complex multifactorial issue for VPT infants, depending on biological, clinical and environmental events (Beaino et al., 2011; Draper et al., 2020; Gibertoni et al., 2020; Linsell et al., 2015; Sentenac, Johnson, et al., 2020; Twilhaar et al., 2018) and BMF practices reflect many of these factors (Bonnet et al., 2019). A systematic review performed to identify the factors for poor cognitive development in children born VPT or VLBW found that the influence of perinatal risk factors on cognitive development appears to diminish over time as other social and environmental factors become more important (Linsell et al., 2015). In our study, although risk (Model 1 the RRs decreased consecutively when we adjusted for perinatal) and additionally for maternal and sociodemographic characteristics (Model 2), never BMF remained associated with a significantly increased risk for suboptimal nonverbal (RR = 1.29) and verbal (RR = 1.45) cognition comparing with those ever breastfed. This result is particularly important for clinical practice highlighting the crucial role of BMF as a minimum standard for newborn care, being a tool to mitigate the adverse neurodevelopmental outcomes among these vulnerable infants. Studies of BMF practices in neonatal units, which are known to be effective for supporting VPT mothers, show there is a large margin for improvement to promote BMF in many countries (Cuttini et al., 2019; Rodrigues et al., 2018; Wilson et al., 2018).

Acting on modifiable risk factors that interfere with optimal neurodevelopment, such as those related to BMF, is important because despite many advances in perinatal care in the last decades, adverse neurodevelopmental outcomes among children born VPT are not declining over time (Pascal et al., 2018; Pierrat et al., 2017; Twilhaar et al., 2018). A meta‐analysis from Blencowe et al. (2013) based on articles with a median birth year of 2000 or later, estimated that 52% of extremely preterm (born at <28 weeks) and 24% of VPT infants (born at 28–31 weeks) develop a certain degree of some neurodevelopmental impairment. A recent meta‐analysis of 71 studies showed a large (0.86 SD) difference in intelligence scores between VPT children and full‐term controls at age 5 years or older, in children born between 1990 and 2008 (Twilhaar et al., 2018). Pascal et al. (2018) estimated a pooled prevalence of cognitive delay of 16.9% (95% CI: 10.4–26.3) in VPT or VLBW infants born after 2006 and evaluated from 18 months CA until 6 years. Pooled estimates of five meta‐analyses affirmed the robust conclusion that VPT birth is associated with a lower IQ, compared with full‐term children (Sentenac, Boutron, et al., 2020).

### 4.1 Strengths and limitations

The strengths of this study include the use of a large population‐based prospective cohort, its geographic spread and range of sociocultural contexts, and standardized data collection tools, with common definitions, across 11 European countries. Although a developmental assessment done by a trained professional is typically considered the gold standard, we used the PARCA‐R, which is a cost‐efficient, accurate, norm‐referenced and standardized measure of children's development at 2 years of age (Johnson et al., 2008, 2019). PARCA‐R has concurrent validity with examiner‐administered developmental tests and excellent test–retest reliability (Cuttini et al., 2012; Johnson et al., 2004, 2008). However, it may be more vulnerable to misclassification error as parents may over or under‐report child achievements.

Another strength of our study is the information available on the duration of BMF until the age of 2 years CA. To the best of our knowledge, there is not any published study from contemporary cohorts of children born VPT (<32 weeks) reporting the association between BMF duration and neurodevelopment outcomes. Most previous studies focused on BMF at time of discharge (any BMF vs. exclusive formula) or during hospitalization period (ever vs. never), all of them with smaller samples sizes and being from single country‐cohorts (Beaino et al., 2011; Gibertoni et al., 2020; Johnson et al., 2011; Rozé et al., 2012). In addition, we used both nonverbal and verbal cognitive development as outcomes, reinforcing the impact of our results. However, for verbal cognition we used a question about vocabulary production, reflecting a very low expressive vocabulary (<10 words), as a validated tool for assessing language was not available for all countries (Zeitlin et al., 2020).

Our study also has other limitations. About 35% of the infants discharged alive were not followed up at 2 years CA. Nevertheless, we were able to compare responders with nonresponders using data at baseline and took into consideration potential bias related to cohort attrition by using inverse probability weighting methods. Sensitivity analyses showed that results were not substantially affected by weighting for nonresponse (data not presented), as observed previously in the EPICE cohort (Bonnet et al., 2019; Seppänen et al., 2020).

In addition, we did not collect detailed infant feeding data, namely, we have no information about the total duration of exclusive BMF, type of formula used and route of BMF administration. The duration of BMF was collected retrospectively when the child was 2 years CA, which may lead to some potential recall bias, although previous studies reported good validity of maternal recall of BMF duration (Amissah et al., 2017). Also, this potential recall bias was mitigated by verifying the data reported by parents with the information on the type of feeding at discharge from hospital abstracted from clinical records, which made it possible to validate partially this information, in accordance with another study already published using EPICE data at 2 years CA (Bonnet et al., 2019).

In the EPICE cohort, 34% of units reported using human bank milk/donor milk for all VPT infants when the mother's own milk was not available (Rodrigues et al., 2021). Thus, within children classified as never breastfed (mother's own milk), some of them may have received donor milk. A sensitivity analysis was carried out considering only children hospitalized in NICUs without human bank milk/donor milk available and we found that the association was even stronger for both non‐verbal and verbal suboptimal cognitive development, compared with those ever breastfed.

Finally, maternal intelligence, which is an important potential confounder of the association between BMF and cognitive development, was not measured. However, in a recent meta‐analysis of studies that controlled for maternal intelligence, BMF remained associated with a gain in performance in IQ testing (Horta et al., 2015).

## 5 CONCLUSION

BMF is associated with both non‐verbal and verbal cognitive development of VPT children at 2 years CA, independently of perinatal and sociodemographic characteristics. The most important conclusion is that any amount of BMF is better than none. Considering that any intervention that has the potential to increase cognitive ability among these most vulnerable children is a significant window of opportunity, our results reinforce the importance of specifically targeted interventions to protect, promote and support BMF in NICUs and after discharge. Further research is needed to understand the impact of BMF initiation and continuation at long‐term neurodevelopmental outcomes in this vulnerable population.

## EPICE (EFFECTIVE PERINATAL INTENSIVE CARE IN EUROPE) RESEARCH GROUP

BELGIUM: Flanders (E. Martens, G. Martens, P. Van Reempts); DENMARK: Eastern Region (K. Boerch, A. Hasselager, L. Huusom, O. Pryds, T. Weber); ESTONIA (L. Toome, H. Varendi); FRANCE: Burgundy, Ile‐de France and Northern Region (P. Y. Ancel, B. Blondel, A. Burguet, P. H. Jarreau, P. Truffert); GERMANY: Hesse (R. F. Maier, B. Misselwitz, S. Schmidt), Saarland (L. Gortner); ITALY: Emilia Romagna (D. Baronciani, G. Gargano), Lazio (R. Agostino, D. DiLallo, F. Franco), Marche (V. Carnielli), M. Cuttini; NETHERLANDS: Eastern & Central (C. Koopman‐Esseboom, A. Van Heijst, J. Nijman); POLAND: Wielkopolska (J. Gadzinowski, J. Mazela); PORTUGAL: Lisbon and Tagus Valley (L. M. Graça, M. C. Machado), Northern region (C. Rodrigues, T. Rodrigues), H. Barros; SWEDEN: Stockholm (A. K. Bonamy, M. Norman, E. Wilson); UK: East Midlands and Yorkshire and Humber (E. Boyle, E. S. Draper, B. N. Manktelow), Northern Region (A. C. Fenton, DWA Milligan); INSERM, Paris (J. Zeitlin, M. Bonet, A. Piedvache).

## CONFLICTS OF INTEREST

The authors declare no conflicts of interest.

## ETHICAL APPROVALS

Informed consent was obtained from all parents or legal representatives included in the follow‐up study. Ethical approvals were obtained in each country as required by national legislation. The European study was also approved by the French Advisory Committee on Use of Health Data in Medical Research and the French National Commission for Data Protection and Liberties.

## AUTHOR CONTRIBUTIONS

Study concept and design were done by Carina Rodrigues, Jennifer Zeitlin and Henrique Barros. All authors (including authors listed in EPICE Research Group) contributed to the acquisition, analysis or interpretation of data. The first draft of the manuscript was done by Carina Rodrigues. Also, the statistical analysis was done by Carina Rodrigues, supervised by Jennifer Zeitlin and Henrique Barros. Lastly, the critical revision of the manuscript for important intellectual content and approval of the final version of the manuscript was done by all authors (including authors listed in EPICE Research Group).

## Supporting information

Supporting information.

## Data Availability

Data available on request due to privacy/ethical restrictions.
