# Supplementary material for: Never‐breastfed children face a higher risk of suboptimal cognition at 2 years of corrected age: A multinational cohort of very preterm children
Source: Matern Child Nutr. 2022 Mar 16;18(3):e13347. doi: 10.1111/mcn.13347 (PMC9218322; doi:10.1111/mcn.13347)
Supplement: Supplementary file 1 — Supporting information. [file MCN-18-e13347-s001.docx]

**SUPPORTING INFORMATION**

**Figure S1. Study population.**

**6792**

**Infants discharged alive from hospital**

Deaths after hospital discharge

and before 2 years CA (n=31)

**4426**

**Infants followed-up**

**at 2 years CA**

**4323**

**Infants followed-up**

**in 18 European regions**

**6761**

**Infants eligible**

**for follow-up**

Losses to follow-up (n=2335)

(of whom 276 in UKN)

Infants from UKN (n=103)

**Infants followed-up by region**

Belgium - Flanders, n= 308

Denmark - Eastern, n= 180

Estonia (whole country), n= 138

France - Burgundy, n= 72

France - Northern, n= 235

France - Ile-de-France, n= 679

Germany - Hesse, n= 368

Germany - Saarland, n= 67

Italy - Emilia, n= 355

Italy - Lazio, n= 296

Italy - Marche, n= 81

Netherlands - East-Central, n= 229

Poland - Wielkopolska, n= 199

Portugal - Lisbon and Tagus Valley, n= 212

Portugal - Northern, n= 196

Sweden - Stockholm, n= 165

UK - East Midlands, n= 253

UK - Yorkshire and Humber, n= 290

CA: Corrected age; UKN: UK Northern Region.

**Table S1. Characteristics associated with loss to follow-up at 2 years corrected age (CA), considering all eligible infants for this study (n=6382).**

|  |  | **Non-responders**  **at 2 years CA**  n=2059 (32.3%) |  | **Responders**  **at 2 years CA**  n=4323 (67.7%) |  |  |
| --- | --- | --- | --- | --- | --- | --- |
| **Characteristics** | **N^a^** | **n (%)** |  | **n (%)** | **p-value^b^** | **p-value^c^** |
| **Mother and pregnancy** |  |  |  |  |  |  |
| **Mother’s age at delivery** | 6364 |  |  |  | <0.001 | <0.001 |
| ≤ 24 years |  | 488 (23.8) |  | 537 (12.4) |  |  |
| 25-34 years |  | 1098 (53.5) |  | 2516 (58.3) |  |  |
| ≥ 35 years |  | 465 (22.7) |  | 1260 (29.2) |  |  |
| **Mother’s country of birth** | 5940 |  |  |  | <0.001 | <0.001 |
| Native-born |  | 1319 (70.5) |  | 3288 (80.8) |  |  |
| Born elsewhere in Europe |  | 117 (6.3) |  | 198 (4.9) |  |  |
| Born outside Europe |  | 434 (23.2) |  | 584 (14.4) |  |  |
| **Parity at delivery** | 6325 |  |  |  | <0.001 | <0.001 |
| 0 previous births |  | 1029 (50.3) |  | 2595 (60.6) |  |  |
| 1 previous birth |  | 532 (26.0) |  | 1044 (24.4) |  |  |
| 2 or more previous birth |  | 485 (23.7) |  | 640 (15.0) |  |  |
| **Type of pregnancy** | 6381 |  |  |  | 0.002 | 0.001 |
| Singleton |  | 1455 (70.7) |  | 2891 (66.9) |  |  |
| Multiple |  | 603 (29.3) |  | 1432 (33.1) |  |  |
| **Infant and neonatal morbidity** |  |  |  |  |  |  |
| **Child sex** | 6381 |  |  |  | 0.320 | 0.581 |
| Male |  | 1118 (54.3) |  | 2291 (53.0) |  |  |
| Female |  | 940 (45.7) |  | 2032 (47.0) |  |  |
| **Gestational age at birth** | 6382 |  |  |  | 0.013 | 0.165 |
| 22-25 weeks |  | 175 (8.5) |  | 326 (7.5) |  |  |
| 26-27 weeks |  | 297 (14.4) |  | 757 (17.5) |  |  |
| 28-29 weeks |  | 553 (26.9) |  | 1153 (26.7) |  |  |
| 30-31 weeks |  | 1034 (50.2) |  | 2087 (48.3) |  |  |
| **Birth weight** | 6382 |  |  |  | 0.160 | 0.492 |
| <1000 grams |  | 545 (26.5) |  | 1217 (28.2) |  |  |
| ≥1000 grams |  | 1514 (73.5) |  | 3106 (71.8) |  |  |
| **SGA** | 6381 |  |  |  | 0.818 | 0.998 |
| <3^rd^ percentile |  | 417 (20.2) |  | 901 (20.8) |  |  |
| 3^rd^ – <10^th^ percentile |  | 240 (11.7) |  | 512 (11.8) |  |  |
| ≥10^th^ percentile |  | 1402 (68.1) |  | 2910 (67.3) |  |  |
| **BPD** | 6241 |  |  |  | 0.233 | 0.153 |
| No |  | 1739 (86.3) |  | 3693 (87.4) |  |  |
| Yes |  | 276 (13.7) |  | 533 (12.6) |  |  |
| **ROP stages III-V** | 6292 |  |  |  | 0.429 | 0.226 |
| No |  | 1951 (96.6) |  | 4112 (96.2) |  |  |
| Yes |  | 68 (3.4) |  | 161 (3.8) |  |  |
| **IVH III-IV / cPVL** | 6306 |  |  |  | 0.089 | 0.119 |
| No |  | 1881 (92.8) |  | 4019 (93.9) |  |  |
| Yes |  | 146 (7.2) |  | 260 (6.1) |  |  |
| **NEC needing surgery or peritoneal drainage** | 6382 |  |  |  | 0.314 | 0.725 |
| No |  | 2020 (98.1) |  | 4256 (98.4) |  |  |
| Yes |  | 39 (1.9) |  | 67 (1.6) |  |  |
| **Any congenital anomaly** | 6380 |  |  |  | 0.696 | 0.412 |
| None |  | 1897 (92.2) |  | 3958 (91.6) |  |  |
| Non-severe |  | 138 (6.7) |  | 315 (7.3) |  |  |
| Severe |  | 23 (1.1) |  | 49 (1.1) |  |  |
| **Perinatal risk** | 6215 |  |  |  | 0.282 | 0.505 |
| Lower |  | 575 (28.8) |  | 1132 (26.9) |  |  |
| Moderate |  | 763 (38.2) |  | 1636 (38.8) |  |  |
| Higher |  | 662 (33.1) |  | 1447 (34.3) |  |  |
| **Breast milk feeding**  **at discharge** | 6218 |  |  |  | <0.001 | <0.001 |
| No |  | 931 (47.6) |  | 1596 (37.4) |  |  |
| Yes |  | 1023 (52.4) |  | 2668 (62.6) |  |  |
| **Country (region(s))** | 6382 |  |  |  | <0.001 | <0.001 |
| Belgium (Flanders) |  | 345 (16.8) |  | 308 (7.1) |  |  |
| Denmark (Eastern) |  | 106 (5.2) |  | 180 (4.2) |  |  |
| Estonia (whole country) |  | 1 (0.1) |  | 138 (3.2) |  |  |
| France (Burgundy, Northern, Ile-de-France) |  | 117 (5.7) |  | 986 (22.8) |  |  |
| Germany (Hesse, Saarland) |  | 222 (10.8) |  | 435 (10.1) |  |  |
| Italy (Emilia, Lazio, Marche) |  | 236 (11.5) |  | 732 (16.9) |  |  |
| Netherlands (East-Central) |  | 101 (4.9) |  | 229 (5.3) |  |  |
| Poland (Wielkopolska) |  | 50 (2.4) |  | 199 (4.6) |  |  |
| Portugal (Lisbon, Northern) |  | 197 (9.6) |  | 408 (9.4) |  |  |
| Sweden (Stockholm) |  | 75 (3.6) |  | 165 (3.8) |  |  |
| UK (East Midlands, Yorkshire and Humber) ^d^ |  | 609 (29.6) |  | 543 (12.6) |  |  |
| ^a^ Proportions were calculated on all cases excluding missing values.  ^b^ Chi-squared test and did not include missing category.  ^c^ Wald test from logistic regression adjusted for country and did not include missing category.  ^d^ UK Northern region was excluded from this analysis.  BPD, bronchopulmonary dysplasia; CA, corrected age; cPV, cystic periventricular leukomalacia; IVH, intraventricular haemorrhage; NEC, necrotising enterocolitis; ROP, retinopathy of prematurity; SGA, small for gestational age. | | | | | | |

**Table S2. Stratified analysis by perinatal risk for non-verbal and verbal cognitive development, according to breast milk feeding (BMF) practices.**

|  | **Lower perinatal risk** | | | **Moderate perinatal risk** | | | **Higher perinatal risk** | | |
| --- | --- | --- | --- | --- | --- | --- | --- | --- | --- |
|  | **Non-verbal cognition** | | | **Non-verbal cognition** | | | **Non-verbal cognition** | | |
|  | **Optimal**  **n (%** ^a^**)** | **Suboptimal**  **n (%** ^a^**)** | **Crude RR** ^b^  **(95% CI)** | **Optimal**  **n (%** ^a^**)** | **Suboptimal**  **n (%** ^a^**)** | **Crude RR** ^b^  **(95% CI)** | **Optimal**  **n (%** ^a^**)** | **Suboptimal**  **n (%** ^a^**)** | **Crude RR** ^b^  **(95% CI)** |
| **BMF initiation** | *(n=1072)* |  |  | *(n=1547)* |  |  | *(n=1354)* |  |  |
| Never breastfed | 189 (89.4) | 21 (10.6) | 1.02 (0.58-1.78) | 256 (84.7) | 45 (15.3) | 1.21 (0.97-1.51) | 247 (68.8) | 101 (31.2) | 1.57 (1.12-2.18) |
| Ever breastfed | 779 (89.9) | 83 (10.1) | Reference | 1086 (86.6) | 160 (13.4) | Reference | 813 (79.8) | 193 (20.2) | Reference |
| **BMF duration** | *(n=1032)* |  |  | *(n=1477)* |  |  | *(n=1270)* |  |  |
| Never breastfed | 189 (89.4) | 21 (10.6) | 0.88 (0.45-1.73) | 256 (84.7) | 45 (15.3) | 1.74 (1.10-2.75) | 247 (68.8) | 101 (31.2) | 1.54 (1.21-1.96) |
| >0 to <2 months | 138 (86.9) | 19 (13.1) | 1.16 (0.64-2.09) | 160 (80.0) | 36 (20.0) | 2.19 (1.24-3.88) | 74 (81.3) | 16 (18.7) | 0.90 (0.52-1.58) |
| 2 to <4 months | 231 (93.6) | 15 (6.4) | 0.53 (0.30-0.92) | 305 (84.5) | 54 (15.5) | 1.64 (1.03-2.61) | 205 (82.5) | 43 (17.5) | 0.85 (0.61-1.20) |
| 4 to <6 months | 136 (91.9) | 11 (8.1) | 0.66 (0.29-1.48) | 162 (89.9) | 19 (10.1) | 1.10 (0.75-1.62) | 171 (79.1) | 42 (20.9) | 1.02 (0.76-1.37) |
| ≥ 6 months | 241 (88.8) | 31 (11.2) | Reference | 401 (90.5) | 39 (9.5) | Reference | 300 (79.5) | 71 (20.5) | Reference |
| *p-for-trend* |  |  | *0.707* |  |  | *0.006* |  |  | *0.071* |
|  | **Verbal cognition** | | | **Verbal cognition** | | | **Verbal cognition** | | |
|  | **Optimal**  **n (%** ^a^**)** | **Suboptimal**  **n (%** ^a^**)** | **Crude RR** ^b^  **(95% CI)** | **Optimal**  **n (%** ^a^**)** | **Suboptimal**  **n (%** ^a^**)** | **Crude RR** ^b^  **(95% CI)** | **Optimal**  **n (%** ^a^**)** | **Suboptimal**  **n (%** ^a^**)** | **Crude RR** ^b^  **(95% CI)** |
| **BMF initiation** | *(n=1093)* |  |  | *(n=1560)* |  |  | *(n=1361)* |  |  |
| Never breastfed | 193 (91.2) | 19 (8.8) | 1.25 (0.68-2.31) | 261 (85.2) | 42 (14.8) | 2.17 (1.23-3.82) | 277 (78.3) | 71 (21.7) | 1.40 (0.92-2.11) |
| Ever breastfed | 828 (93.8) | 53 (6.2) | Reference | 1164 (92.6) | 93 (7.4) | Reference | 857 (84.0) | 156 (16.0) | Reference |
| **BMF duration** | *(n=1048)* |  |  | *(n=1484)* |  |  | *(n=1269)* |  |  |
| Never breastfed | 193 (91.2) | 19 (8.8) | 1.01 (0.44-2.30) | 261 (85.2) | 42 (14.8) | 3.06 (1.39-6.73) | 2778 (78.3) | 71 (21.7) | 1.42 (0.94-2.14) |
| >0 to <2 months | 153 (92.0) | 14 (8.0) | 1.19 (0.53-2.65) | 180 (89.9) | 19 (10.1) | 2.04 (0.82-5.05) | 74 (82.2) | 14 (17.8) | 1.08 (0.55-2.14) |
| 2 to <4 months | 234 (96.4) | 9 (3.6) | 0.48 (0.11-2.19) | 331 (92.0) | 28 (8.0) | 1.50 (0.81-2.79) | 209 (84.3) | 40 (15.7) | 1.02 (0.72-1.43) |
| 4 to <6 months | 140 (95.1) | 7 (4.9) | 0.63 (0.26-1.48) | 173 (94.8) | 11 (5.2) | 1.15 (0.52-2.57) | 188 (90.0) | 23 (10.0) | 0.66 (0.34-1.28) |
| ≥ 6 months | 260 (91.7) | 19 (8.3) | Reference | 414 (94.5) | 25 (5.5) | Reference | 317 (84.7) | 56 (15.3) | Reference |
| *p-for-trend* |  |  | *0.533* |  |  | *0.029* |  |  | *0.158* |
| BMF, Breast milk feeding (mother’s own milk); RR, Risk ratio.  ^a^ Proportions were calculated using inverse probability weights to account for non-response bias; ^b^ Risk ratios were derived from the weighted sample (inverse probability of participating at the 2 years CA follow-up), multilevel mixed-effects generalised linear regression models with random intercepts at the country and mother level. | | | | | | | | | |

**Table S3. Stratified analysis by mother´s education level for non-verbal and verbal cognitive development, according to breast milk feeding (BMF) practices.**

|  | **Low educational level** | | | **Intermediate educational level** | | | **High educational level** | | |
| --- | --- | --- | --- | --- | --- | --- | --- | --- | --- |
|  | **Non-verbal cognition** | | | **Non-verbal cognition** | | | **Non-verbal cognition** | | |
|  | **Optimal**  **n (%** ^a^**)** | **Suboptimal**  **n (%** ^a^**)** | **Crude RR** ^b^  **(95% CI)** | **Optimal**  **n (%** ^a^**)** | **Suboptimal**  **n (%** ^a^**)** | **Crude RR** ^b^  **(95% CI)** | **Optimal**  **n (%** ^a^**)** | **Suboptimal**  **n (%** ^a^**)** | **Crude RR** ^b^  **(95% CI)** |
| **BMF initiation** | *(n=768)* |  |  | *(n=1683)* |  |  | *(n=1514)* |  |  |
| Never breastfed | 209 (81.2) | 47 (18.8) | 1.13 (0.78-1.62) | 293 (77.7) | 75 (22.3) | 1.55 (1.01-2.38) | 171 (82.1) | 37 (17.9) | 1.52 (0.96-2.43) |
| Ever breastfed | 431 (83.4) | 81 (16.6) | Reference | 1124 (85.0) | 191 (15.0) | Reference | 1144 (86.9) | 162 (13.1) | Reference |
| **BMF duration** | *(n=723)* |  |  | *(n=1604)* |  |  | *(n=1448)* |  |  |
| Never breastfed | 209 (81.2) | 47 (18.8) | 1.30 (0.81-2.07) | 293 (77.7) | 75 (22.3) | 1.74 (1.05-2.87) | 171 (82.1) | 37 (17.9) | 1.47 (0.93-2.31) |
| >0 to <2 months | 80 (84.2) | 15 (15.8) | 1.11 (0.66-1.87) | 183 (82.9) | 35 (17.1) | 1.24 (0.58-2.66) | 107 (83.5) | 18 (16.5) | 1.24 (0.62-2.49) |
| 2 to <4 months | 139 (83.7) | 27 (16.3) | 1.16 (0.63-2.13) | 348 (86.9) | 51 (13.1) | 0.92 (0.63-1.35) | 264 (87.3) | 36 (12.7) | 0.93 (0.62-1.38) |
| 4 to <6 months | 66 (88.5) | 8 (11.5) | 0.76 (0.32-1.82) | 189 (82.6) | 39 (17.4) | 1.30 (0.84-2.00) | 229 (89.8) | 26 (10.2) | 0.75 (0.34-1.63) |
| ≥ 6 months | 114 (86.3) | 18 (13.7) | Reference | 342 (86.9) | 49 (13.1) | Reference | 487 (86.3) | 73 (13.7) | Reference |
| *p-for-trend* |  |  | *0.101* |  |  | *0.070* |  |  | *0.037* |
|  | **Verbal cognition** | | | **Verbal cognition** | | | **Verbal cognition** | | |
|  | **Optimal**  **n (%** ^a^**)** | **Suboptimal**  **n (%** ^a^**)** | **Crude RR** ^b^  **(95% CI)** | **Optimal**  **n (%** ^a^**)** | **Suboptimal**  **n (%** ^a^**)** | **Crude RR** ^b^  **(95% CI)** | **Optimal**  **n (%** ^a^**)** | **Suboptimal**  **n (%** ^a^**)** | **Crude RR** ^b^  **(95% CI)** |
| **BMF initiation** | *(n=783)* |  |  | *(n=1700)* |  |  | *(n=1519)* |  |  |
| Never breastfed | 207 (80.8) | 50 (19.2) | 1.70 (1.02-2.82) | 321 (85.8) | 51 (14.2) | 1.76 (1.09-2.84) | 184 (88.8) | 22 (11.2) | 1.33 (0.73-2.42) |
| Ever breastfed | 460 (86.1) | 66 (13.9) | Reference | 1208 (91.4) | 120 (8.6) | Reference | 1204 (91.6) | 109 (8.4) | Reference |
| **BMF duration** | *(n=735)* |  |  | *(n=1607)* |  |  | *(n=1453)* |  |  |
| Never breastfed | 207 (80.8) | 50 (19.2) | 1.79 (0.71-4.51) | 321 (85.8) | 51 (14.2) | 2.02 (1.20-3.41) | 184 (88.8) | 22 (11.2) | 1.26 (0.71-2.24) |
| >0 to <2 months | 84 (83.9) | 15 (16.1) | 1.41 (0.59-3.35) | 203 (92.1) | 20 (7.9) | 1.08 (0.60-1.93) | 120 (91.7) | 8 (8.3) | 0.81 (0.42-1.53) |
| 2 to <4 months | 154 (92.6) | 13 (7.4) | 0.66 (0.23-1.91) | 359 (90.8) | 37 (9.2) | 1.15 (0.78-1.70) | 274 (91.2) | 26 (8.8) | 0.98 (0.64-1.52) |
| 4 to <6 months | 67 (90.9) | 7 (9.1) | 0.71 (0.22-2.38) | 205 (92.0) | 20 (8.0) | 1.23 (0.78-1.96) | 243 (94.2) | 16 (5.8) | 0.60 (0.21-1.69) |
| ≥ 6 months | 121 (87.1) | 17 (12.9) | Reference | 361 (92.4) | 30 (7.6) | Reference | 512 (91.4) | 48 (8.6) | Reference |
| *p-for-trend* |  |  | *0.098* |  |  | *0.030* |  |  | *0.473* |
| BMF, Breast milk feeding (mother’s own milk); RR, Risk ratio.  ^a^ Proportions were calculated using inverse probability weights to account for non-response bias; ^b^ Risk ratios were derived from the weighted sample (inverse probability of participating at the 2 years CA follow-up), multilevel mixed-effects generalised linear regression models with random intercepts at the country and mother level. | | | | | | | | | |
